# Multi-stakeholder perspective of courier service quality in B2C e-commerce

**DOI:** 10.1371/journal.pone.0251728

**Published:** 2021-05-21

**Authors:** Aleksandra Gulc

**Affiliations:** Faculty of Engineering Management, Bialystok University of Technology, Bialystok, Poland; Univerza v Mariboru, SLOVENIA

## Abstract

Under conditions of the rapidly developing e-commerce sector especially during pandemic, ensuring high quality of courier service is essential both for clients, as well as courier companies. However, the literature lacks research linking the perspective of clients and organization in the context of courier service quality. The study aims to identify the factors determining courier service quality, their functions and interrelationships in business-to-customer (B2C) e-commerce. The main effect of research is the relational model, which is an original and complex approach to courier service quality considering the multi-stakeholder perspective of an online shop, a courier company and an e-customer. Apart from scientific contribution, the model can be used into managerial practice to formulate the recommendations for e-commerce and courier service sector. The research process involved using the quantitative method (electronic surveys conducted among e-shops and e-clients) and the qualitative method (in-depth-interviews carried out among courier enterprises). Finally, based on the empirical research results, the structural analysis was used to develop the model. As a result, the following groups of factors were distinguished that determine the quality of courier services: crucial, determinant, result, autonomous and external factors.

## Introduction

The courier, express and parcel (CEP) market has reached impressive growth rates worldwide from the past decade. E-commerce is among the major drivers of the CEP market development generating significant revenues, especially in the last year, during the pandemic caused by a coronavirus. Physical distancing, business lockdown and other confinement measures have accelerated growing trends in e-commerce. With consumers facing pandemic-related constraints worldwide, Internet users turned to online shopping more frequently and ordered even essential goods [1]. The situation resulted in the rise of business-to-consumer (B2C) sales and affected business-to-business (B2B) e-commerce. By the end of May 2020, online orders were doubled year-on-year in North America and increased by 50% in Europe [2]. Before the pandemic, the value of the global courier market reached EUR 330.3 billion in 2019, and according to forecasts, it will reach EUR 400 billion by 2024, which means an increase of 8–10% annually in subsequent years [3]. The dynamics of the CEP market in Poland, reaching 15% annually, is one of the highest in Europe. The value of Polish courier market amounted to EUR 2,3 billion in 2020, noting the increase by 20% compared to 2019. At the same period, the volume of shipments increased by 34,9% reaching the level of 814 million parcels. Since 2015 till 2020, the number of parcels in Polish market has increased by 163% [4]. Poland is among eight European countries (Germany, Great Britain, France, Spain, Italy, the Netherlands, and Belgium), which generate 76% of the European GDP and 80% of the total revenues from courier service in Europe. Although CEP market has been growing dynamically in Poland, it is still relatively young and prospective as it represents only 3.5% of the European market in terms of value and 4.8% in terms of volume [5]. CEP market in Poland is very diversified and saturated both by global integrators offering complex logistics service, as well as small and medium companies specialising in urban delivery. Courier market in Poland is dominated by ten largest operators, whose share amounted to 97.6% of total volume and 94.6% of total revenue in 2019. Among the leaders there are mainly global integrators like DPD, DHL, UPS, FedEx, Geis and only two Polish enterprises: the national postal operator–Poczta Polska S.A. and one company founded in Poland with foreign capital–InPost [6]. It is worth emphasising that Poland is one of the most dynamically developing European markets in terms of out-of-home delivery just after Germany, France, the UK and Italy. The number of PUDO (PickUp Drop Off) points in Poland increased by 70% at the turn of 2020/2021 compared to the middle of 2019, while it grew by 40% in Europe in the same period [4]. The development of the CEP market in Poland has undoubtedly been influenced by the growing popularity of e-shopping, facilitated by better internet access and the growing customer confidence in online shopping. As a result, the structure of the client segment has gradually changed, and B2C services have become dominant in terms of the shipment volume. In 2020, B2C e-commerce orders accounted for 76% of all shipments, while B2B transactions represented 18,7% of the market, and C2X – 4,7%. In the lockdown period, as many as 67% of consumers used courier services. As a result, the courier market value grew by 30%, much more than 20% in 2019. The seasonal peaks in sales, as well as the coronavirus pandemic and the lockdown, contributed to the increasing number of Poles buying online [4,7]. Considering e-clients choice, courier service is the most preferred form of parcel delivery, the quality of which affects customer opinions about e-shops. As a result, effective logistics has become a crucial factor in gaining a competitive advantage and success for online shops [7]. Moreover, the specificity of the e-commerce, particularly including the individual requirements of e-consumers, forced courier operators to offer dedicated solutions such as mobile applications to track&trace the delivery, bots or chat-bots instead of traditional customer service and pick-up-drop-off networks [8–14]. According to expert forecasts, the high dynamics of CEP market growth in Poland will be maintained up to the value of EUR 3,6 billion in 2023, which means the increase of as much as 514 percent in three years. Considering the volume of shipments, the analysts predict that courier companies will pick up 1,31 billion parcels in 2023 (61% more than in 2020) [4].

The rapid development of e-commerce and increasing customer expectations make service quality improvement an essential objective for courier companies [4–14]. However, the literature considering courier service quality is limited. The research carried out so far did not reflect the specificity of the e-commerce branch, as they usually focused on recipients of courier services (individuals or business customers) omitting the sender—an online shop and a courier company [15]. However, many authors emphasised the need for further research on service quality integrating both perspectives: customers (external quality) and organisations (internal quality). Such efforts would shed new light on service quality and suggest key directions for quality improvement [16–22]. This paper presents the final part of a complex study, aiming to fill such gaps. Based on the review and analysis of literature concerning courier service quality in the context of e-commerce development, the following research gap was identified: the lack of a comprehensive approach concerning determinants of courier service quality in the e-commerce sector from the perspective of three stakeholder groups: e-shops, courier companies and e-clients. Based on the identified research gap, the research problem was defined: which factors and their interrelations impact courier service quality in the B2C e-commerce sector?

This research mainly aims to identify functions and relationships between factors that determine the courier service quality in the B2C e-commerce sector considering the multi-stakeholder perspective of online shops, courier companies and e-customers. The main result of this study is the relational model that reflects the factors affecting the courier service quality and their interrelations, i.e., the crucial and other factors that perform various functions in the analysed system. As a result, five groups of factors that determine courier service quality were distinguished: crucial factors, determinants, results, autonomous and external factors. Among 12 analysed factors, the most important was an efficient and fast order processing (SERV), which turned out to be the crucial factor. The group of determinant factors classified: the responsiveness of a courier company to reported problems (RES), easy contact with the courier company (CON), and efficient communication between courier company employees and clients (online shops and e-customers) (COM). The group of result factors included the timeliness of delivery (TIM), the effectiveness of delivery (EFF), and positive relationships and the customer experience with courier service (REL). The factor compliance and completeness of the order (COMP) was classified as an autonomous factor. In contrast, external factors included the lack of damage to the shipment (DAM), cultured and courteous behaviour of courier company employees (CUL), flexibility in the choice or change of date and place of delivery (FLE), and the choice of the form of parcel sending or delivering (FOR). The relational model can be used to support the implementation of improvement actions concerning service quality in courier enterprises.

### Literature review concerning the courier service quality

Customer-perceived service quality is often used as an indicator for measuring business performance and market position affecting the competitive advantage [23]. The perceived service quality is defined as the level, to which a provided service matches customer expectations [24–26], finally affecting customer loyalty [27,28]. A part of research on perceived service quality studies concentrates on identifying service quality determinants in various sectors and branches. Parasuraman, Zeithaml and Berry developed a service quality model called the gap model [29], which became the frequently cited and modified model by authors in the service sector [15]. It presents four quality gaps arising in a company during the service delivery and the fifth gap during the contact with a client. The model includes ten following service quality determinants perceived by clients: reliability, responsiveness, competence, access, courtesy, communication, credibility, security, understanding, and tangibles [29]. The gap model authors developed the measuring scale of the perceived service quality called SERVQUAL, which included a reduced number of service quality dimensions: tangibles, responsiveness, reliability, empathy, and assurance [30]. Although the scale was criticised by many authors and, usually, adjusted to the specificity of a particular branch, it became the most used scale in the service sector [15].

Although aspects of the courier service quality are crucial and trendy due to e-commerce development, scientific literature has only several studies on the topic. Most of analysed studies focused on measuring courier service quality using the modified SERVQUAL method and some criteria of service quality were adapted to the specific nature of the courier service [31–35]. According to Tabassum and Badiuddin study, the customers perceived that courier service companies were empathetic and reliable, while their responsiveness, which entails a willingness to help the customers and deliver prompt service, was ranked last [31]. Liu and Liu defined and examined slightly different criteria of service quality. Reliability was also the most important dimension, while perception was the least significant. Moreover, security and empathy were rated the lowest, while safety and perception received the highest scores [32]. Similarly to Liu and Liu, the research conducted by Yee and Daud indicated that reliability was the most important for customers, while empathy concerned them the least. The regression analysis results showed that tangibility, reliability and assurance impacted customer satisfaction, while empathy and responsiveness had no significant effect [33]. Similarly to above mentioned authors, Ho et al. study aimed to identify the quality dimensions of courier services that contribute to customer satisfaction [34]. However, the authors applied quality dimensions consistent with the logistic scale of services (LSQ), previously developed by other authors [36]. The multiple regression analysis showed that in the context of the achieved satisfaction, the condition of order was a priority dimension for customers using courier services, thereby it replaced the timeliness, which was usually the most important critical dimension for the service quality in other research. It was also found that the quality of information had a major impact on customer satisfaction with courier services, which is in line with the results obtained by other researchers [37,38]. Valaei et al. developed the measurement scale called CouQual, which was based on the modified SERVQUAL method, according to the specificity of courier services. Based on the research results, the developed model of perceived quality indicated that timeliness, safety and convenience positively impacted the perceived service quality, while accuracy and tangibility had no significant impact [35].

In contrast to the previously discussed studies, a completely different approach was proposed by Yu et al. who considered the perspective of the service provider in terms of meeting customer requirements and needs in a two-stage method for improving the service quality. The method was developed based on the quality gap model, Quality Functional Deployment method and the fuzzy set theory. The courier industry’s research results have shown the most important aspects that a service enterprise should highlight to meet customer requirements. Studies by Yu et al. were innovative in terms of their subject matter, as they were concerned with identifying quality determinants inside a service company to eliminate four quality gaps [39]. The pilot study carried out by Gulc focused on the present and future expectations of clients towards courier service. Respondents assessed that the most critical criteria in the future would be the time of delivery, trust, flexibility and teletechnologies, while the price would be less important [40]. Further empirical research conducted by Gulc aimed to identify and classify the key factors which determined the courier service quality perceived by e-customers using the exploratory factor analysis. According to the results, the most important dimension affecting the courier service quality was the reliability manifesting as timeliness, a successful delivery attempt, the completeness of delivery and the lack of damage to the shipment, while visual identification and social responsibility were the least important [41]. The research by Ejdys and Gulc aimed to examine relationships between five constructs concerning courier service. The main result was the model presenting the relationship between trust in courier service, perceived service quality and future intentions to use the courier service. The results confirmed statistically significant relationships between the variables or the ease of use and the trust in service, the usefulness and the trust in service, the trust in service and the service quality, and, finally, the service quality and the future intention to use the service. The developed scale to measure the usefulness, the ease of use and customer trust in the context of courier service research was the most crucial contribution to the methodology [42].

To sum up the literature review, the authors of available studies have not developed the universal set of factors determining the courier service quality, therefore it is difficult to identify consistent conclusions. The factors were often selected according to the differentiated and specific criteria, such as the type of provided service, the segment of customers and the geographical and cultural context. Most of research indicated that reliability was the most significant factor of courier service quality [31–33], while responsiveness was considered as the least one [31,33]. In case of empathy, the opinions were not coherent [31,33]. So far, the studies were often fragmentary and situational, as they focused on the narrow group of respondents, the selected region of a particular country or only one of the quality criteria [31–35]. Moreover, the scientific research conducted so far was mainly concentrated on identifying criteria/attributes/factors affecting the courier service quality and the methods of evaluating them, omitting the existing relationship between the factors. The research on the courier service quality considered only one perspective, usually clients of courier services (individuals or business customers) [31–35,39–42] omitting the perspective of the sender—an online shop and a courier company. Considering the conclusions of analysed research on service quality, the article aims to analyse courier service quality from a multi-shareholder perspective, as it allows to formulate comprehensive and novel conclusions towards future directions of service quality.

## Material and methods

To construct the relational model, empirical research focused on three main courier service stakeholders in the e-commerce branch. Therefore, the research process included five stages: quantitative research of online shops and, later, e-customers using courier service, qualitative research of courier enterprises, structural analysis, and finally, the development of the relational model. The empirical part of the research was conducted in 2019, and the final two parts in 2020. The study was non-interventional in nature and did not require permission from the Ethics Committee. All respondents agreed to participate in the study and their consent was written (quantitative study) and verbal (qualitative study). The surveys and interviews were anonymous. The qualitative research used the Computer Assisted Web Interview (CAWI) technique in the form of an electronic questionnaire used to survey e-customers and e-shops in Poland. The survey focused on Polish online shops selling various products. The respondents were asked to assess the impact of factors on the courier service quality using the 7-level Likert scale. The list of factors, which is presented in Table 1, based on SERVQUAL scale was prepared as a result of literature review. In 2018, according to the Central Register and Information on Business, the number of e-shops amounted to 28.9 thousand. The minimum sample size was 384, assuming a confidence level of 0.95 (1-α) and a maximum permissible error of 5% calculated for the general population of about 28 thousand online shops. An electronic questionnaire was used to conduct confidential interviews; it was distributed between January and March 2019; 405 questionnaires were fully filled and returned, so the research results could be generalised for the whole population. The research sample was differentiated in terms of the size and duration of business activity. The majority of online shops participating in the study were small enterprises with fewer than ten employees (94.3%), while the smallest groups included large enterprises with over 250 employees (0.5%) and medium-sized enterprises with 49 to 249 employees (5.2%). The sample structure confirmed the e-commerce market trends in terms of the duration of business activity, in which about 60% of shops were operating for less than ten years. The medium share was recorded by shops operating for six to ten years (24.2%) and the least by those with over ten years of market activity (14.8%).

**Table 1:** List of factors determining the courier service quality.

Dimension	Factors
tangibility	modern transport fleet
	expanded and well-equipped operational network (sorting centres, branches, pick-up/drop-off points)
	modern and functional ICT technologies (applications for shipment monitoring and tracking, mobile applications)
	modern technical solutions (parcel machines, pick-up/drop-off points, drones as couriers)
	ecological technical solutions (electric cars/bikes, drones, ecological packaging)
	interesting and attractive information and promotional materials
	promotional materials (leaflets, advertising slogans, website, hotline)
	aesthetic and neat appearance of the courier
	distinctive company logo and uniform colouring
reliability	attractive prices and discounts
	timeliness of delivery
	effectiveness of delivery
	compliance and completeness of the order
	lack of damage to the shipment
	quick refund upon return of the consignment
	correct contract documentation
	transparent procedures, documents and standards of service
	simplicity of making an order
	positive experience with courier service
	positive opinions of other clients
assurance	positive image and brand of the courier company
	courier company experience
	knowledge and competences of courier company employees
	cultured and courteous behaviour of courier company employees
	transactions security
	easy contact with courier company
	efficient communication between courier company employees and clients
	trust in courier company
responsiveness	accurate and clear information on the terms of service
	efficient and fast order processing
	efficient handling of returns
	responsiveness of courier company to reported problems
	flexibility in the choice or change of date and place of delivery
empathy	individualisation of service (offering a convenient time of service, date and method of payment)
	giving the customer full attention
	taking care of the customer’s interests and protecting them
	involvement of the courier company in social campaigns
	compliance with business ethics principles by the courier company
other	wide range of additional services
	service availability (convenient location of branches and drop-off/pick-up points, convenient working hours)
	various scope and range of provided services
	flexibility in the choice or change of date and place of delivery (delivery at home/work, dispatch or collection in other places such as branch, kiosk, parcel machine)

Source: Own study based on [31–43].

The second part of research considered customers in Poland who had used courier service in the last three years to order products over the Internet. Due to the lack of data on the number of clients using courier services in Poland, the general population was assumed to be the number of persons ordering or buying goods or services via the Internet for private use. In 2018, according to the Central Statistical Office (GUS), the number amounted to 14,094,377 persons. In total, 594 fully completed questionnaires were received, making it possible to generalise the results (the minimum sample size amounted to 384, assuming a confidence level of 0.95 and a maximum permissible error of 5%). An electronic survey was distributed between April and May 2019. The respondents were asked to assess the impact of factors on the courier service quality using the 7-level Likert scale (the list of factors is presented in Appendix).The share of women in the study was 52% (309 persons), and 48% of respondents were men (285 persons). Among the respondents, 31.5% were 36–45 years of age, 22.2% were 26–35, and 15.8% were 46–55. The age groups below 25 and over 55 constituted about 15% of the respondents each. Both online shops and e-clients taking part in the survey represented all regions of Poland and the sample size distribution corresponded to the general population. Based on survey results, the exploratory factor analysis was used to indicate the correlation between variables and classified factors into theoretical constructs [43].

Later, qualitative research in the form of in-depth interviews was conducted in ten major courier enterprises. One part of interviews assessed the impact of factors on the service quality by the experts–the managers from courier companies (the same list of factors was used as in the previous research among online shops and e-customers).

A cross-impact analysis, otherwise known as the structural analysis, was used to identify the key factors influencing the courier service quality from the multi-stakeholder perspective in order to formulate conclusions and recommendations considering service quality. The author decided to carry on the research based on this method, as it provides an opportunity for a thorough presentation of the system under study. Identification of key variables influencing the analysed system is necessary to evolve and implement appropriate policies and strategies. Therefore, structural analysis can be used in forecasting and decision making process to achieve the desired objectives [44].

The main aim of the structural analysis is to detect and understand the mutual interactions among variables of interest and categorize them in terms of driving and dependence power. Finally, all the variables are classified into specified clusters with diversified functions in analysed system [45,46]. The advantage of the cross-impact analysis is the ability to identify relationships between variables which mutual influences are not obvious and may remain unrecognised even by experts in the field [47].

The structural analysis can be made using the MICMAC method developed by Michel Godet and François Bourse. The method is based on the algorithm using the multiplication properties of matrices [45,48,49]. The first stage of its implementation consists of making an inventory of all variables and/or factors, internal or external, that characterize the system. Next stage includes the description of mutual relationships between variables. The experts assess if there is the interaction between the pair of factors and determine its strength (low, medium, high or potential) using a four-stage scale. By analysing the relationships between the factors, a direct and indirect impact graphs are drawn. Finally, the last stage involves the classification of factors influencing the research area into the following clusters [47]:

- crucial factors—characterised by high impact and high dependency on other factors; they require particular attention and research due to instability;
- aim factors—represent possible aims of the analysed system; they are more dependent on other factors and are impact by them rather than vice versa;
- result factors—are characterised by low impact and high dependency on other factors; are particularly susceptible to changes in crucial factors;
- determinant factors (drivers and brakes)—have a powerful influence on the system and low impact on other factors; may be considered as a driving or braking force but are difficult to control;
- regulatory and supplementary factors—have little impact on the system but may help achieve strategic objectives;
- external factors—have less impact on the system compared to determinants but more than autonomous variables;
- autonomous factors—have the least impact on changes in the system as a whole.

**Fig 1:**
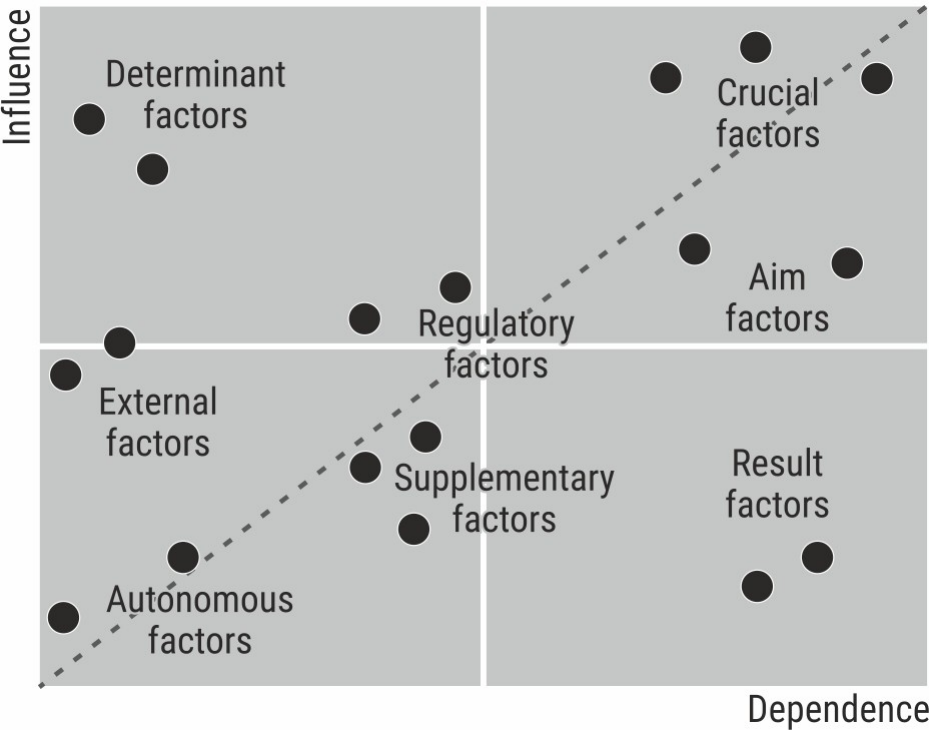
Arrangement of factors impacting the analysed research area. Source: [45,47].

The groups of mentioned above factors are presented on the influence-dependence chart in Fig 1.

Structural analysis, which had been primarily used as one of the tool in futures studies, has experienced since the middle of the 1980’s an increasing number of applications in various fields concerning businesses as well as on society-related topics considering different aspects of management [47–58] including quality management [52–55].

As this research was based on the structural analysis using the MICMAC application, it was conducted in three stages. The first stage involved the compilation of the list of factors determining the courier service quality from the perspective of the three stakeholders involved in the courier service provision (e-shops, individual recipients, and courier companies). Based on quantitative and qualitative research results, the final list of factors was prepared, which included the highest factors rated by at least two groups of respondents (the mean value was above 6.0) (Table 2).

**Table 2:** List of key factors determining the courier service quality.

Acronym	Factor
**TIM**	timeliness of delivery
**EFF**	effectiveness of delivery
**DAM**	lack of damage to the shipment
**CUL**	cultured and courteous behaviour of courier company employees
**COM**	efficient communication between courier company employees and clients
**RES**	responsiveness of courier company to reported problems
**SERV**	efficient and fast order processing
**COMP**	compliance and completeness of the order
**REL**	positive relationships and the customer experience with courier service
**CON**	easy contact with the courier company
**FLE**	flexibility in the choice or change of date and place of service
**FOR**	choosing the form of sending or delivery

Source: Own study.

Factors assessed by only one respondent group were not included in the structural analysis aiming to keep the final number of factors limited and the matrix transparent and legible as well as to ensure an appropriate duration of the expert panel.

The second stage of the structural analysis aimed to determine whether the factors impacted on other factors in the analysed area and what was the extent of the impact. The impact strength of factors was assessed by experts using a four-stage scale, in which 0 meant “no impact”, 1—“weak impact”, 2—“medium impact” and 3—“high impact”. Sixteen experts participated in the panel: representatives with academic experience in logistics, managers from courier companies, owners of online shops, and experienced customers using courier services. As a result, a direct impact matrix was created based on a direct influence matrix individually filled in by the experts. The resultant direct impact matrix of factors influencing the courier service quality is presented in Table 3.

**Table 3:** Impact strength of twelve factors.

	TIM	EFF	DAM	CUL	COM	RES	SERV	COMP	REL	CON	FLE	FOR
**TIM**	0	3	0	0	0	0	3	0	3	0	0	0
**EFF**	3	0	0	0	0	2	3	0	3	0	0	0
**DAM**	1	3	0	0	0	0	3	3	3	0	0	0
**CUL**	1	2	0	0	2	1	1	0	3	2	2	0
**COM**	3	2	0	2	0	2	3	0	2	3	1	1
**RES**	3	3	0	0	3	0	3	1	3	2	2	0
**SERV**	3	3	1	0	1	3	0	2	3	1	0	2
**COMP**	2	3	0	0	0	0	3	0	2	0	0	0
**REL**	0	0	0	0	2	1	0	0	0	2	0	0
**CON**	2	3	0	1	2	2	3	0	3	0	3	1
**FLE**	3	3	0	0	0	0	3	0	3	0	0	2
**FOR**	2	2	0	0	0	0	3	0	3	0	3	0

Source: Own study.

The experts participating in the study identified 144 relationships between factors (variables). In 72 cases, the dominant value was zero, meaning no relationship between the variables. In 13 cases, weak relationships were found, and in 23 cases, the relationships were of medium strength. Strong relationships between the variables were identified in 36 cases.

## Results

Table 4 presents the summary results of the calculations concerning the impact strength and direct relationships. The results indicate the following factors having a strong direct impact on the factors: SERV—the efficient and fast order processing, RES—the responsiveness of courier company to reported problems, CON—easy contact with the courier company and COM—the efficient communication between courier company employees, an e-shop, and an e-customer. The following factors were most dependent on the other factors: REL—positive relationships and the customer experience with courier service, SERV—efficient and fast order processing, EFF—the efficiency of delivery, and TIM—the timeliness of delivery. The lack of damage to the shipment (DAM) and the cultured and courteous behaviour of courier company employees (CUL) had the lowest dependence on other factors. Positive relationships and the customer experience with courier service (REL) had the least direct influence on other factors.

**Table 4:** Summary of direct interaction strengths between factors in the structural analysis.

Acronym	Factor	Total impact strength	Total dependence strength
**TIM**	timeliness of delivery	9	23
**EFF**	effectiveness of delivery	11	27
**DAM**	lack of damage to the shipment	13	1
**CUL**	cultured and courteous behaviour of courier company employees	14	3
**COM**	efficient communication between courier company employees and clients	19	10
**RES**	responsiveness of courier company to reported problems	20	11
**SERV**	efficient and fast order processing	19	28
**COMP**	compliance and completeness of the order	10	6
**REL**	positive relationships and the customer experience with courier service	5	31
**CON**	easy contact with the courier company	20	10
**FLE**	flexibility in the choice or change of date and place of delivery	14	11
**FOR**	choice of the form of parcel sending or delivering	13	6

Source: Own study.

**Fig 2:**
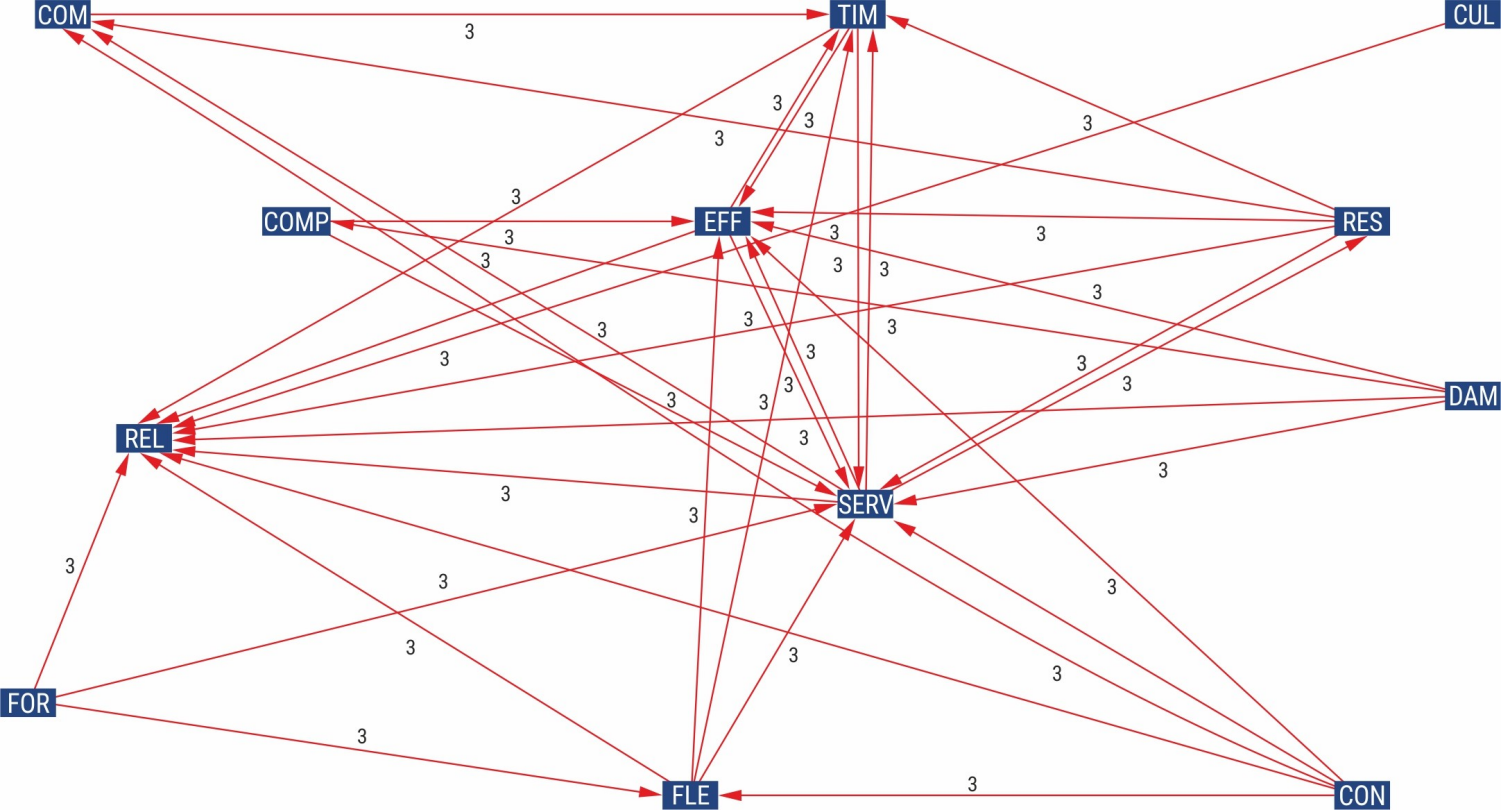
Direct impact graph. Source: Own study.

In the next part of the analysis, a graph showing the strong direct impact of factors was created using the MICMAC programme (Fig 2). It should be noted that almost all factors had strong correlations with several other factors (marked “3” on the graph), apart from the cultured and courteous behaviour of courier company employees (CUL), which had a high impact only on positive relationships and the customer experience with courier service (REL).

The analysis of the graph presented in Fig 2 indicates that the four factors have the strongest relationships with other factors, including the efficient and fast order processing (SERV), the timeliness of delivery (TIM), the effectiveness of delivery (EFF), and positive relationships and the customer experience with courier service (REL). It is worth noting that the efficient and fast order processing (SERV) is highly dependent on and strongly influenced by other factors.

**Fig 3:**
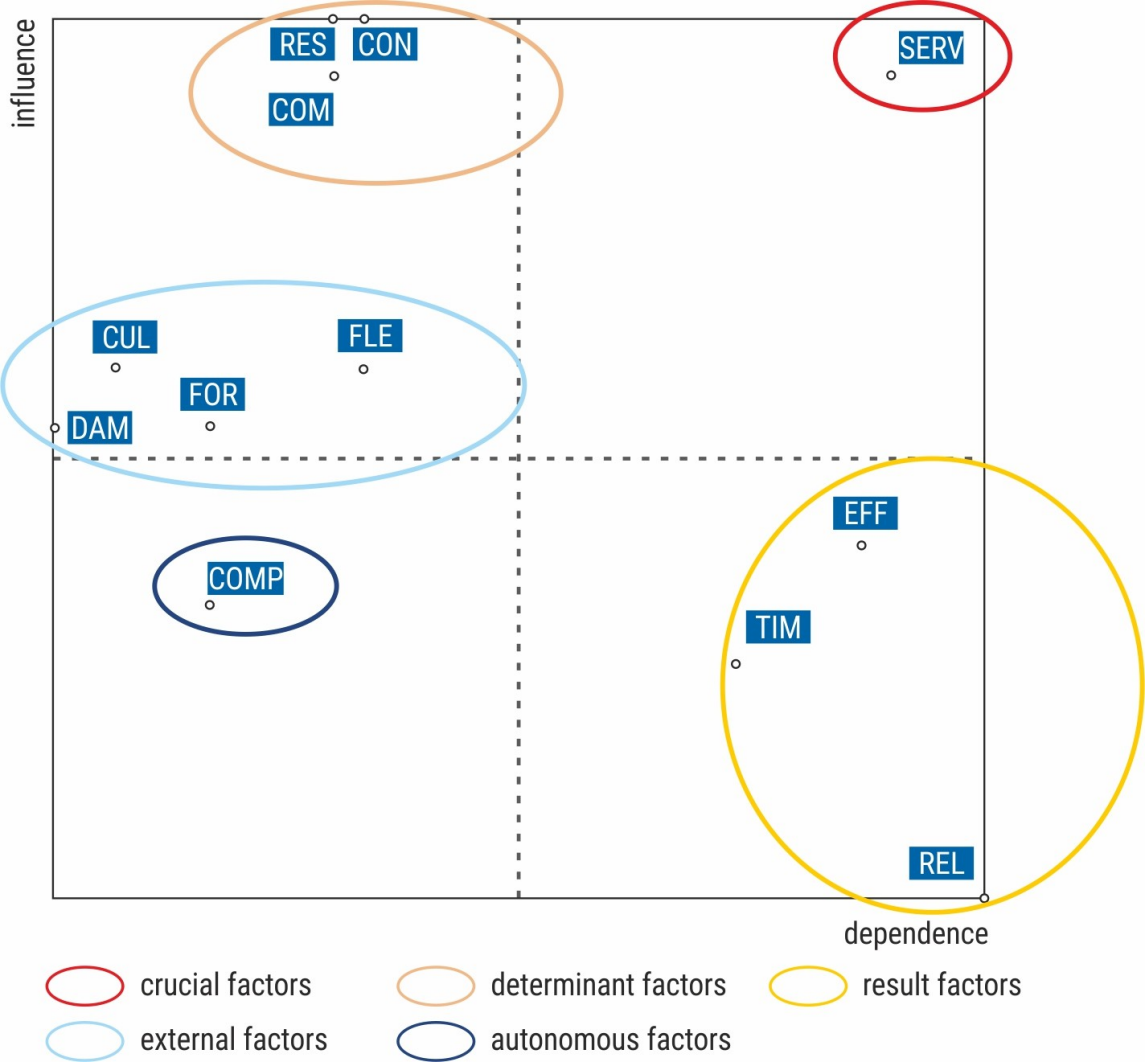
Influence–dependence graph. Source: Own study.

During the next stage of the structural analysis, the influence–dependence chart was drawn using the MICMAC programme (Fig 3). As a result, the following groups of factors determining the courier service quality were distinguished: the crucial factor, determinant factors, result factors, and autonomous and external factors. The structural analysis did not reveal other groups of factors often indicated in the literature, including regulatory, aim, and supplementary factors.

The most important factor in the examined system was the efficient and fast order processing (SERV), which turned out to be the crucial factor combining strong influence with a high dependency on other factors. The group of determinant factors included: responsiveness of courier company to reported problems (RES), easy contact with the courier company (CON), and efficient communication between courier company employees and clients (online shops and e-customers) (COM). Particular attention should be paid to determinant factors having a high impact on the system. They may be considered a driving or braking force, but they are also difficult to control. The group of result factors with low impact and high dependence on other factors included: the timeliness of delivery (TIM), the effectiveness of delivery (EFF), and positive relationships and the customer experience with courier service (REL). The factor compliance and completeness of the order (COMP) was classified as an autonomous factor with a low impact on the system and the smallest dependency. The last group were external factors having a less important impact on the system compared to the determinants, which was higher than that of the autonomous factors. There were four factors in this group: the lack of damage to the shipment (DAM), cultured and courteous behaviour of courier company employees (CUL), flexibility in the choice or change of date and place of delivery (FLE), and the choice of the form of parcel sending or delivering (FOR).

**Fig 4:**
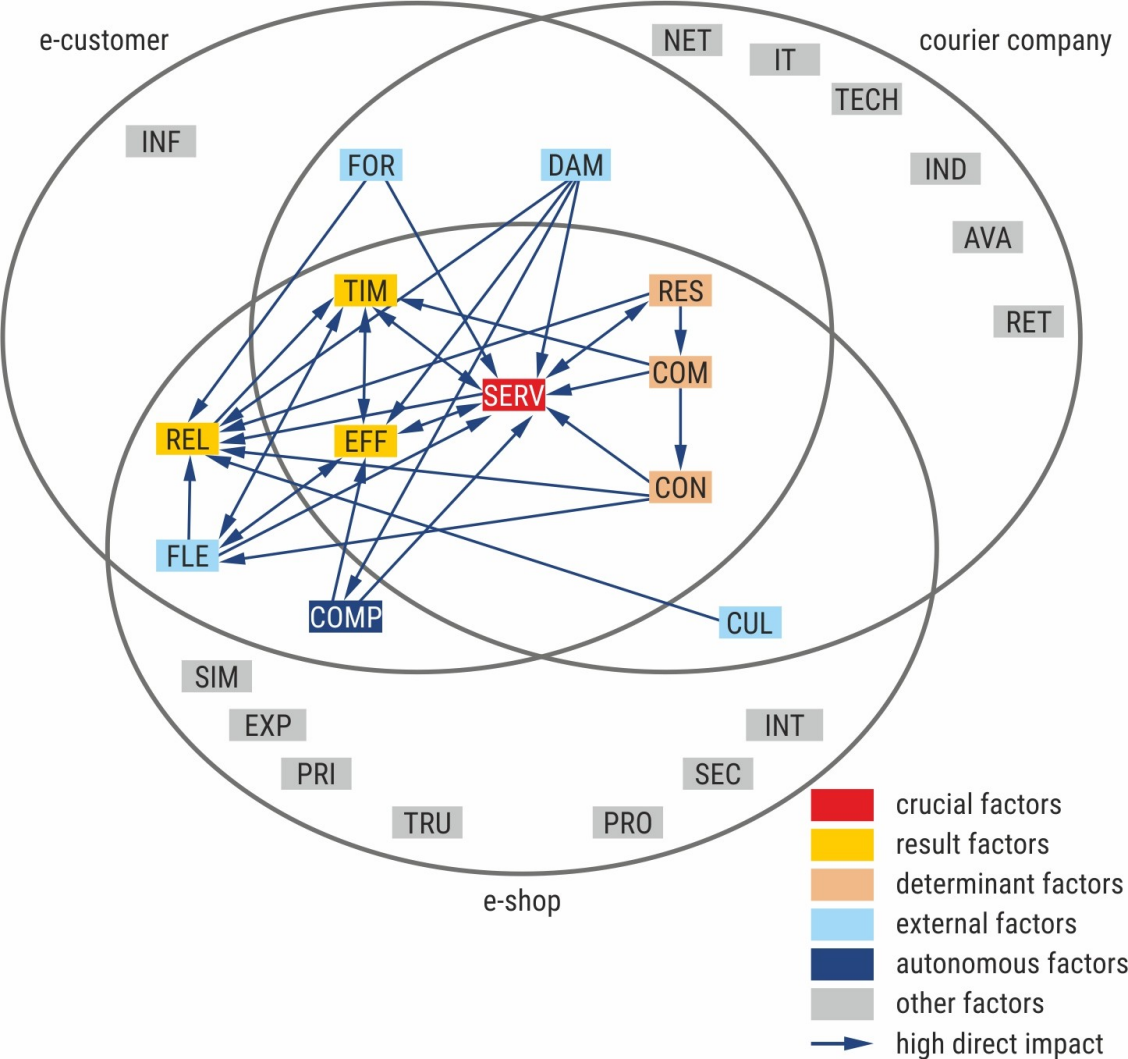
Relational model of courier service quality in the B2C e-commerce sector. Source: Own study.

The final result of all previous research stages was developing the relational model of the courier service quality in the e-commerce sector considering B2C relations shown in Fig 4. The model presents the factors determining the courier service quality, strong direct relationships between the factors and the functions performed by the factors in the analysed system, from the perspective of three groups of stakeholders: an e-shop, an e-customer, and a courier company.

According to all analysed stakeholders, efficient and fast order processing (SERV) is crucial for the courier service quality. Order processing includes all stages of the courier service process, starting from the acceptance of an e-customer order in an online shop until the delivery of the parcel to the e-customer. Efficient and fast order processing depends on many factors and influences the timeliness of delivery (TIM), the effectiveness of delivery (EFF), and the responsiveness of courier company to reported problems (RES). All three entities indicated two result factors—the timeliness of delivery (TIM) and the effectiveness of delivery (EFF)—which depend on other factors and impact the system less. The result determinants, which have a strong impact on other factors and less dependency, are aspects related to ensuring efficient communication between courier company employees and clients (online shops and e-customers) (COM), the easy contact with the courier company (CON), and the responsiveness of courier company to reported problems (RES).

As far as the relationship between an e-customer and a courier company is concerned, two additional factors in the context of service quality are indicated: the choice of the form of parcel sending or delivering (FOR), and the lack of damage to the shipment (DAM). Considering the courier service quality in the analysed system, these factors play the role of external factors, so they are independent of other factors and have little influence on the system.

From the perspective of a courier company and an e-shop, only one external factor is independent of other factors—the cultured and courteous behaviour of courier company employees (CUL). This factor strongly influences positive relationships and customer experience with courier service (REL).

According to an e-customer and an e-shop, three additional factors are important in the context of the perception of courier service quality: positive relationships and the customer experience with courier service (REL), the flexibility in the choice or change of date and place of delivery (FLE), and the compliance and completeness of the order (COMP). It is worth highlighting the critical role of positive relationships and the customer experience with courier service (REL), as this factor is strongly dependent on some factors while having no influence on others. On the other hand, the flexibility in the choice or change of date and place of delivery (FLE) serves as an external factor, which has little impact on the system and is rarely dependent on other factors. This factor affects the timeliness of delivery (TIM), the effectiveness of delivery (EFF), and the relationships between stakeholders, and at the same time, it depends on the easy contact with the courier company (CON). The compliance and completeness of the order (COMP) is an autonomous factor which has the least influence on the courier service quality and the least dependence on other factors.

The model also presents factors specific to each analysed entity and not included in the structural analysis. It should be stressed that each group indicated completely different factors. According to courier companies, factors influencing the service quality were mainly tangible and connected with technical, technological and infrastructure aspects of service, including the extensive and well-equipped operating network (NET), information and communication technologies (IT), and modern technical solutions (TECH). Other factors were related with customer needs on the service availability (AVA) understood as a convenient location of branches and pick-up-drop-off points as well as convenient working hours, but also the quick refund upon return of the consignment (RET), and the individualisation of the service ensuring convenient delivery time and form of payment (IND). In the case of online shops, the following factors were important: the security of transactions (SEC), the trust in the courier company (TRU), the experience and credibility of the courier company (EXP), and the protection of the client interests (INT). Moreover, the courier service quality was also perceived in the context of attractive pricing and discount policy (PRI). Online shops also appreciated the functionality of the service, meaning the simplicity of ordering (SIM) and the transparent procedures, documents and standards of service (PRO). E-customers paid particular attention to accurate and clear information on the conditions of service provision (INF).

## Discussion

A part of the obtained results was consistent with conclusions reached by other authors. Most previous studies also indicated the timeliness of delivery (TIM) as a priority dimension for customers using courier services [32,34,38–41]. Liu and Liu included the compliance and completeness of the order (COMP) among the most important factors determining the courier service quality analysed in this study [32]. It should be emphasised that the study revealed one of the most important group of factors—determinant factors—that require particular attention as they may be considered as a driving or braking force but are difficult to control. These factors concerned the empathy dimension including the efficient communication between stakeholders (COM), the positive relationships and the customer experience with courier service (REL), and easy contact with the courier company (CON). This result was in accordance with conclusions by Saura et al. [27] and Tabassum and Badiuddin [31] but opposed the outcomes by Liu and Liu and Ho et al. [32,34]. Unlike previously analysed research, the relational model presented in this paper includes many novel elements. First, the study presents the complex approach towards the courier service quality in the context of B2C e-commerce specificity joining the perspective of three entities: an e-shop, a courier company and an e-customer. Also, the application of the structural analysis not only allowed indicating the factors determining the courier service quality but also identifying their functions in the analysed area and direct interrelationships. Therefore, the main contribution to the management theory is the relational model reflecting the relationships between factors and their functions in the context of the service quality in B2C e-commerce.

The research results also contribute to the managerial practice in the field of courier services. Based on the developed model, the author formulated the recommendations for the improvement of the courier service quality in the e-commerce sector. The priority area of the service quality should be the efficient and fast order processing, as it depends on many different factors related to the technical and functional quality, and at the same time influences other factors determining the courier service quality. The study revealed that the aspects concerning communication, contact, and the responsiveness of courier company employees were particularly important in the context of ensuring a high courier service quality. These factors highly impact other factors, including the timeliness and effectiveness of delivery, but also the efficient and fast order processing, and, consequently, affect the positive relationships with customers. Therefore, companies providing courier service should try and develop interpersonal and communication skills of employees directly working with clients. Particular attention should be paid the most important factors of the courier service quality from the perspective of all stakeholders—the timeliness of delivery and effectiveness of delivery—which strongly depend on other factors and, therefore, are susceptive to changes. To ensure on-time and efficient delivery of parcels, courier companies should continue to invest in the development of their infrastructure (in particular, the network of pick-up-drop-off points) and modern information technology providing access to mobile and personalised service. It is imperative to implement solutions aimed at the further automation of courier service and the use of artificial intelligence to shorten the time of service delivery. Under conditions of increasing competitiveness in the courier market, the priority strategic objective is to retain and attract new customers. The study results confirmed that the positive relationships and the customer experience with courier service also affect the service quality; therefore, courier companies should offer added value to customers.

The author is aware of the study limitations in terms of evaluating the results and effectiveness of applying the relational model in courier service companies. Another limitation is related to the B2C segment of e-commerce and the research sample, which only involved Polish respondents, making the conducted research national.

The research findings suggest several directions for future efforts. The other segments of e-commerce, like B2B or C2X service, would be the potential areas for future research on determinants of courier service. It would also be interesting to conduct similar research in different countries of the world and identify cultural differences affecting the perception of the courier service quality. The dynamic development of courier service and modern technologies focused on end-consumers indicate that similar research should be repeated in the future to observe if the determinants of service quality change with time. Further research would consider the m-commerce sector as the current global trend resulting from further utilisation of mobile and wireless technologies and clients shifting from e-commerce to m-commerce [59].

## Conclusion

This study contributes to the scientific research literature on e-commerce and courier service quality. It proposes a relational model presenting factors determining courier service quality, their functions, and relationships in B2C e-commerce. To the best of the author’s knowledge, this research is the first effort to study service quality using a comprehensive approach that considers a perspective of multiple stakeholders engaged in courier service in B2C e-commerce: e-shops, courier companies, and e-clients. The main study findings are the groups of factors determining the courier service quality: crucial, determinant, result, external, and autonomous. Among these five groups, crucial, result and determinant factors should be considered in the context of the future directions of the service quality improvement. The efficient and fast order processing, which impacts many other factors and depends on them, appeared to be the crucial factor for the courier service quality. Result factors, including the timeliness and effectiveness of delivery, and the positive relationships and the customer experience with courier service, are especially dependent on other factors. At the same time, they are particularly susceptible to changes in crucial factors. The study also revealed the importance of client service, including the aspects of communication, responsiveness and contact with clients. These determinant factors may be considered as a driving or braking force; therefore, they should be particularly examined in the context of the service quality in B2C e-commerce. It is worth emphasising that the relational model also presents the determinants of the courier service quality, which are specific to a particular group of analysed entities evolved in B2C e-commerce. Courier companies indicate technical and technological solutions, which corresponds to a tendency to increasing the automation of courier service in the future. E-customers pay attention to accurate and clear information on the conditions of service provision. According to e-shops, the following aspects are important: trust, service functionality, experience, and reliability of courier company, the security of transactions, and protection of customer interests. At the same time, the courier service quality is still perceived from the point of view of attractive pricing and discount policy. To sum up, the relational model can be used as a tool supporting the implementation of improvement actions concerning the service quality in courier enterprises as it reflects the key areas determining the service quality.
